# Supplementary material for: Provider-led community antiretroviral therapy distribution in Malawi: Retrospective cohort study of retention, viral load suppression and costs
Source: PLOS Glob Public Health. 2023 Sep 28;3(9):e0002081. doi: 10.1371/journal.pgph.0002081 (PMC10538660; doi:10.1371/journal.pgph.0002081)
Supplement: S1 Table — (DOCX) [file pgph.0002081.s001.docx]

| Model of care | HR | P-Value | 95% CI | aHR* | P-Value | 95% CI |
| --- | --- | --- | --- | --- | --- | --- |
|  |  |  |  |  |  |  |
| Hub | 1 (ref) |  |  | 1 (ref) |  |  |
| CAD | 1.01 | 0.950 | 0.64-1.50 | 1.05 | 0.838 | 0.66-1.66 |

*aHR adjusted for age, sex and district.
