# Supplementary material for: Provider-led community antiretroviral therapy distribution in Malawi: Retrospective cohort study of retention, viral load suppression and costs
Source: PLOS Glob Public Health. 2023 Sep 28;3(9):e0002081. doi: 10.1371/journal.pgph.0002081 (PMC10538660; doi:10.1371/journal.pgph.0002081)
Supplement: S2 Table — (DOCX) [file pgph.0002081.s002.docx]

| Model of care | OR | P-Value | 95% CI | aOR* | P-Value | 95% CI |
| --- | --- | --- | --- | --- | --- | --- |
| Hub | 1 (ref) |  |  | 1 (ref) |  |  |
| CAD | 1.34 | 0.542 | 0.52-3.45 | 1.24 | 0.662 | 0.47-3.25 |

*aOR adjusted for age, sex, and district.
