# Supplementary material for: Provider-led community antiretroviral therapy distribution in Malawi: Retrospective cohort study of retention, viral load suppression and costs
Source: PLOS Glob Public Health. 2023 Sep 28;3(9):e0002081. doi: 10.1371/journal.pgph.0002081 (PMC10538660; doi:10.1371/journal.pgph.0002081)
Supplement: S1 Checklist — (DOCX) [file pgph.0002081.s003.docx]

STROBE Statement—checklist of items that should be included in reports of observational studies

|  | Item No. | Recommendation | Page  No. | Relevant text from manuscript |
| --- | --- | --- | --- | --- |
| **Title and abstract** | 1 | (*a*) Indicate the study’s design with a commonly used term in the title or the abstract | 1 | “Retrospective cohort study” |
|  |  | (*b*) Provide in the abstract an informative and balanced summary of what was done and what was found | 2 | “We selected an equal number of clients in CAD and facility-based care who were aged >13 years, had an undetectable viral load (VL) result in the last year and were stable on first-line ART for ≥1 year. We compared retention in care (alive and no period of ≥60 days without ART) using Kaplan-Meier survival analysis and Cox regression and maintenance of VL suppression (<1,000 copies/mL) during follow-up using logistic regression. We also compared costs (in US$) from the health system and client perspectives for the two models of care. Data were collected in October and November 2020”  “Retention in care did not differ significantly between clients in CAD (89.4% retained) and facility-based care (89.3%), p=0.95. No significant difference in maintenance of VL suppression were observed between CAD and facility-based care (aOR: 1.24, 95% CI: 0.47-3.20, p=0.70). CAD resulted in slightly higher health system costs than facility-based care: $118/year vs. $108/year per person accessing care; and $133/year vs. $122/year per person retained in care” |
| Introduction | | | |  |
| Background/rationale | 2 | Explain the scientific background and rationale for the investigation being reported | 4 | “Despite widespread implementation of CAD across sub-Saharan Africa, evidence regarding its effectiveness and cost-effectiveness in improving retention and viral load (VL) suppression is limited, outcomes have varied and none have been reported from Malawi. An early systematic review of studies from sub-Saharan Africa suggested that retention in care is higher in provider- and community-led CAD than in standard facility-based care (6), but two later randomized trials in Zimbabwe and Lesotho found that retention in care and VL suppression did not differ significantly between health facility-based care, community-led CAD, and provider-led CAD (7,8). Compared to standard facility-based care, CAD models had higher health system cost per client retained in care at 12 months in Zambia (9).” |
| Objectives | 3 | State specific objectives, including any prespecified hypotheses | 5 | “we sought to evaluate the impact of provider-led CAD services on retention and VL suppression in Malawi. We also assess differences in cost between provider-led CAD services and facility-based care from a health systems and individual client perspective.” |
| Methods | | | |  |
| Study design | 4 | Present key elements of study design early in the paper | 5 | “Comparing retrospective cohort data from clients receiving provider-led CAD and clients receiving facility-based care, we sought to evaluate the impact of provider-led CAD services on retention and VL suppression in Malawi” |
| Setting | 5 | Describe the setting, locations, and relevant dates, including periods of recruitment, exposure, follow-up, and data collection |  | “Data were collected in October-November 2020, from two ‘hub’ health facilities in Chikwawa and two in Lilongwe, and 20 associated CAD ‘spoke’ sites. We selected all the clients registered in CAD between January 2019 (when CAD implementation began) and June 2019, and collected data from their first visit through August 2020 to allow for 14 months of observation time.”  “We selected hub controls chronologically, starting with those who had visited the facility in January 2019, and proceeding until the required number of clients was reached” |
| Participants | 6 | (*a*) *Cohort study*—Give the eligibility criteria, and the sources and methods of selection of participants. Describe methods of follow-up  *Case-control study*—Give the eligibility criteria, and the sources and methods of case ascertainment and control selection. Give the rationale for the choice of cases and controls  *Cross-sectional study*—Give the eligibility criteria, and the sources and methods of selection of participants | 6 | “We selected all the clients registered in CAD between January 2019 (when CAD implementation began) and June 2019, and collected data from their first visit through August 2020 to allow for 14 months of observation time”  “We selected hub controls chronologically, starting with those who had visited the facility in January 2019, and proceeding until the required number of clients was reached. Clinic visits were used as a method of follow-up during the 14 months of follow-up time.”  Eligibility criteria: “being stable on first-line ART for more than 12 months, registered at a hub site, ready to disclose HIV status to other members of the CAD spoke, undetectable result of the last VL test (within 12 months), and age 13 years or older” |
|  |  | (*b*) *Cohort study*—For matched studies, give matching criteria and number of exposed and unexposed  *Case-control study*—For matched studies, give matching criteria and the number of controls per case |  |  |
| Variables | 7 | Clearly define all outcomes, exposures, predictors, potential confounders, and effect modifiers. Give diagnostic criteria, if applicable | 7 | “*Adverse outcome:* recorded to have died, defaulted or stopped ART during follow-up. For individuals with multiple adverse outcomes recorded, the earliest outcome was used for all analyses. For example, if a client had defaulted, then returned to care, and subsequently died, they were classified as having defaulted.  *Defaulted:* per national guidelines, defaulting from care was defined as being overdue for an ART refill appointment and estimated to have run out of ARVs for 2 months or longer, based on the quantity of ARVs dispensed at the last visit (10).  *Viral load outcomes:* VL results were categorized according to national HIV guidelines: high VL (≥1000 copies/ml); low level viremia (200-999 copies/mL); or suppressed VL (undetectable-199 copies/mL). For logistic regression analyses, we applied a commonly-used binary VL outcome: <1000 copies/mL and ≥1000 copies/mL (11). VL results were only included from samples taken at least 6 months and no more than 18 months after enrollment into CAD or from the first visit in the study period for controls. We extended the period for VL results from 14 to 18 months due to the infrequency of VL testing during the study period.” |
| Data sources/ measurement | 8* | For each variable of interest, give sources of data and details of methods of assessment (measurement). Describe comparability of assessment methods if there is more than one group | 8 | “We compared viral load outcomes between the two models of care using chi-square tests and logistic regression. We used Kaplan-Meier survival analysis methods to compare retention in care at CAD spokes versus hub facilities over the 14 months of follow-up. Each client started contributing person-time to the analysis from the day that they were in enrolled into CAD or the earliest day that they visited the hub facility between January 2019 and June 2019. Follow-up time of clients who transferred to another health facility and of those who remained in care at the end of the follow-up period was censored on their last recorded visit day within the 14 months follow up period. We used Cox regression to produce crude and adjusted hazard ratios (aHR) of experiencing an adverse outcome, adjusting for sex, age and district.”  “For health systems cost per CAD spoke clinic day, we include all additional activities required to successfully implement a CAD visit, including use of a vehicle to and from the CAD (average of 32km round-trip per CAD visit), and the cost of a day of the full clinical team’s services. The total costs were divided by the number of visiting clients to each CAD included in the study to determine the cost per individual client CAD visit.” |
| Bias | 9 | Describe any efforts to address potential sources of bias | 16 | “As is common with observational studies, our results may be prone to bias as clients enrolled in CAD are selected based on specific characteristics, such as being clinically stable, which cannot be completely adjusted for in statistical analyses. However, great effort was put into ensuring that the eligibility criteria used for enrolment into CAD is adhered to during selection of controls through a strict study enrolment protocol that was double checked at data collection and at analysis.”  We did multivariable analyses to address confounding bias “We used Cox regression to produce crude and adjusted hazard ratios (aHR) of experiencing an adverse outcome, adjusting for sex, age and district” |
| Study size | 10 | Explain how the study size was arrived at | 6 | “We selected all the clients registered in CAD between January 2019 (when CAD implementation began) and June 2019, and collected data from their first visit through August 2020 to allow for 14 months of observation time” |

Continued on next page

| Quantitative variables | 11 | Explain how quantitative variables were handled in the analyses. If applicable, describe which groupings were chosen and why | 8 | “We compared viral load outcomes between the two models of care using chi-square tests and logistic regression. We used Kaplan-Meier survival analysis methods to compare retention in care at CAD spokes versus hub facilities over the 14 months of follow-up. Each client started contributing person-time to the analysis from the day that they were in enrolled into CAD or the earliest day that they visited the hub facility between January 2019 and June 2019. Follow-up time of clients who transferred to another health facility and of those who remained in care at the end of the follow-up period was censored on their last recorded visit day within the 14 months follow up period. We used Cox regression to produce crude and adjusted hazard ratios (aHR) of experiencing an adverse outcome, adjusting for sex, age and district.” |
| --- | --- | --- | --- | --- |
| Statistical methods | 12 | (*a*) Describe all statistical methods, including those used to control for confounding | 8 | “We compared viral load outcomes between the two models of care using chi-square tests and logistic regression. We used Kaplan-Meier survival analysis methods to compare retention in care at CAD spokes versus hub facilities over the 14 months of follow-up. Each client started contributing person-time to the analysis from the day that they were in enrolled into CAD or the earliest day that they visited the hub facility between January 2019 and June 2019. Follow-up time of clients who transferred to another health facility and of those who remained in care at the end of the follow-up period was censored on their last recorded visit day within the 14 months follow up period. We used Cox regression to produce crude and adjusted hazard ratios (aHR) of experiencing an adverse outcome, adjusting for sex, age and district.” |
|  |  | (*b*) Describe any methods used to examine subgroups and interactions |  |  |
|  |  | (*c*) Explain how missing data were addressed | 8 | “We assessed the extent of missing data in the sample by key outcome variables (retention and VL suppression). Only VL suppression had missing data (31%) but variations were not statistically significant by arm of intervention (CAD/hub) (p= 0.09). All observations with missing data on VL suppression were excluded from analysis when computing ORs for VL suppression.” |
|  |  | (*d*) *Cohort study*—If applicable, explain how loss to follow-up was addressed  *Case-control study*—If applicable, explain how matching of cases and controls was addressed  *Cross-sectional study*—If applicable, describe analytical methods taking account of sampling strategy | 7 | Loss to follow up is an outcome of interest and is defined as defaulting from care “Defaulted: per national guidelines, defaulting from care was defined as being overdue for an ART refill appointment and estimated to have run out of ARVs for 2 months or longer, based on the quantity of ARVs dispensed at the last visit (10).” In other words, loss to follow up was considered an outcome of interest. |
|  |  | (*e*) Describe any sensitivity analyses |  |  |
| Results | | | | |
| Participants | 13* | (a) Report numbers of individuals at each stage of study—eg numbers potentially eligible, examined for eligibility, confirmed eligible, included in the study, completing follow-up, and analysed | 9 | “We collected data on 700 ART clients, 350 from provider-led CAD and 350 from facility care (Table 2)” |
|  |  | (b) Give reasons for non-participation at each stage |  |  |
|  |  | (c) Consider use of a flow diagram |  |  |
| Descriptive data | 14* | (a) Give characteristics of study participants (eg demographic, clinical, social) and information on exposures and potential confounders | 9 | “We collected data on 700 ART clients, 350 from provider-led CAD and 350 from facility care (Table 2)” Table 2 provides detailed info. |
|  |  |  |  |  |
|  |  | (b) Indicate number of participants with missing data for each variable of interest | 8 | “We assessed the extent of missing data in the sample by key outcome variables and covariates (retention, VL suppression, sex, age and district). Only VL suppression had missing data (21%) but missing data did not vary by arm of intervention (CAD/hub) (p= 0.09).” |
|  |  | (c) *Cohort study*—Summarise follow-up time (eg, average and total amount) | 11 | “The average follow-up time was 11.6 months for clients at CAD spokes and 11.1 months at hub facilities.” |
| Outcome data | 15* | *Cohort study*—Report numbers of outcome events or summary measures over time | 10 | “At the end of the 14-months of follow-up, approximately 1% of participants had died, 10% had defaulted, and 5% had transferred to another health facility.” |
|  |  | *Case-control study—*Report numbers in each exposure category, or summary measures of exposure |  |  |
|  |  | *Cross-sectional study—*Report numbers of outcome events or summary measures |  |  |
| Main results | 16 | (*a*) Give unadjusted estimates and, if applicable, confounder-adjusted estimates and their precision (eg, 95% confidence interval). Make clear which confounders were adjusted for and why they were included | 11-13 | “The cumulative probability of retention in care over the follow-up period was not significantly different between clients in provider-led CAD and facility-based HIV care (88.4% vs. 88.3%; p-value 0.95) (Figure 1).”  “Controlling for sex, age, and district of residence, the risk of experiencing an adverse outcome was similar for clients in provider-led CAD and facility-based care (aHR: 1.05, 95%CI: 0.66-1.66, P-value: 0.80)”  “After adjusting for sex, duration on ART, age, and district of residence, there was no significant difference in prevalence of VL <1,000 copies/ml between clients in CAD (97%) and in hub care (96%): aOR 1.24, 95% CI 0.47-3.26, p-value 0.66.”  “Health system cost estimates for provider-led CAD were $118 per person receiving care per year and $108 per person per year in facility-based care. Cost for individual clients was lower in provider-led CAD ($3.17 per person per year) than facility-based care ($11.44 per person per year).” |
|  |  | (*b*) Report category boundaries when continuous variables were categorized |  |  |
|  |  | (*c*) If relevant, consider translating estimates of relative risk into absolute risk for a meaningful time period |  |  |

Continued on next page

| Other analyses | 17 | Report other analyses done—eg analyses of subgroups and interactions, and sensitivity analyses |  |  |
| --- | --- | --- | --- | --- |
| Discussion | | | | |
| Key results | 18 | Summarise key results with reference to study objectives | 14 | “we found that retention in care and viral suppression outcomes were similar in provider-led CAD as compared to facility-based care. While health system cost per person provided care and per person retained in care were 9% higher in provider-led CAD, cost for 12-months of HIV care incurred by clients was 72% lower in CAD than in facility-based care.” |
| Limitations | 19 | Discuss limitations of the study, taking into account sources of potential bias or imprecision. Discuss both direction and magnitude of any potential bias | 15 | “As is common with observational studies, our results may be prone to bias as clients enrolled in CAD are selected based on specific characteristics, such as being clinically stable, which cannot be completely adjusted for in statistical analyses. However, great effort was put into ensuring that the eligibility criteria used for enrollment into CAD is adhered to during selection of controls.”  “A limitation of our cost analysis is that we did not incorporate travel costs for clients (cost of transportation to/from facility-based care) as many clients walk, use their own bicycle or make informal arrangements that are not directly tied to financial cost” |
| Interpretation | 20 | Give a cautious overall interpretation of results considering objectives, limitations, multiplicity of analyses, results from similar studies, and other relevant evidence | 16 | “Provider-led CAD services in Malawi had excellent one-year retention and VL suppression results that were similar to facility-based care. CAD was associated with a small increase in the financial costs to the health system but substantial savings for clients, which may benefit longer term engagement in care and ART outcomes.” |
| Generalisability | 21 | Discuss the generalisability (external validity) of the study results |  |  |
| Other information | |  | | |
| Funding | 22 | Give the source of funding and the role of the funders for the present study and, if applicable, for the original study on which the present article is based | 16 | “We are grateful for funding by PEPFAR through USAID that allowed Partners in Hope to implement the provider-led CAD program and to collect the data for this analysis.” |

*Give information separately for cases and controls in case-control studies and, if applicable, for exposed and unexposed groups in cohort and cross-sectional studies.

**Note:** An Explanation and Elaboration article discusses each checklist item and gives methodological background and published examples of transparent reporting. The STROBE checklist is best used in conjunction with this article (freely available on the Web sites of PLoS Medicine at http://www.plosmedicine.org/, Annals of Internal Medicine at http://www.annals.org/, and Epidemiology at http://www.epidem.com/). Information on the STROBE Initiative is available at www.strobe-statement.org.
