# Supplementary material for: Provider-led community antiretroviral therapy distribution in Malawi: Retrospective cohort study of retention, viral load suppression and costs
Source: PLOS Glob Public Health. 2023 Sep 28;3(9):e0002081. doi: 10.1371/journal.pgph.0002081 (PMC10538660; doi:10.1371/journal.pgph.0002081)
Supplement: Attachment — Submitted filename: PGPH-D-23-00966 Response to Reviewers.docx [file pgph.0002081.s005.docx]

Academic Editor
PLOS Global Public Health

RE: PGPH-D-23-00966: Provider-led community Antiretroviral Therapy distribution in Malawi: Retrospective cohort study of retention, viral load suppression, and costs.

Thank you to the Editor and the Reviewers for their thoughtful and thorough reviews. We have responded to each point below and have made changes to the manuscript based on reviewers’ suggestions. We believe that the paper is strengthened as a result, and can be accepted for publication.

**Journal Requirements:**

**1. Please review your reference list to; ensure that it is complete and correct. If you have cited papers that have been retracted, please include the rationale for doing so in the manuscript text, or remove these references and replace them with relevant current references. Any changes to the reference list should be mentioned in the rebuttal letter that accompanies your revised manuscript. If you need to cite a retracted article, indicate the article’s retracted status in the References list and also include a citation and full reference for the retraction notice.**

***Response***

The references have been checked. We cite no retracted papers.

**2. In the Funding Information you indicated that no funding was received. Please revise the Funding Information field to reflect funding received.**

***Response***

Thank you very much for this suggestion. We have now changed accordingly.

**3. In the online submission form, you indicated that "The data for this manuscript will be made available to the to the editorial team if need
be upon request through the corresponding author". All PLOS journals now require all data underlying the findings described in their manuscript to be freely available to other researchers, either 1. In a public repository, 2. Within the manuscript itself, or 3. Uploaded as supplementary information.
This policy applies to all data except where public deposition would breach compliance with the protocol approved by your research ethics board. If your data cannot be made publicly available for ethical or legal reasons (e.g., public availability would compromise patient privacy), please explain your reasons by return email and your exemption request will be escalated to the editor for approval. Your exemption request will be handled independently and will not hold up the peer review process, but will need to be resolved should your manuscript be accepted for publication. One of the Editorial team will then be in touch if there are any issues.**

***Response***

While we understand the journal’s requirement of making all data related to this manuscript publicly available, we are unfortunately unable to comply with this requirement due to the reasons highlighted below

1.	Data contain potentially identifying or sensitive participant information

Even though the data for this manuscript are de-identified, the information that the study was conducted at Partners in Hope’s supported sites may draw attention to individual clients attending ART clinics and health care workers at the health facilities and associated CAD spokes.

2.	Who has imposed the restriction of publicly sharing the manuscript data?

The Malawi’s National Health Sciences Research Committee approved our protocol in which under the ethical considerations section on protection of human subjects’ privacy and confidentiality, we stated that participant information will not be released without written permission from the researchers, except as necessary for review, monitoring, and/or auditing.

3.	Provide non-author contact information for a data access committee, ethics committee, or other institutional body to which data requests may be sent

Prof Joep J. van Oosterhout can be contacted through his email address (joep@pihmalawi.com) for any data requests. He will then initiate a communication with the National Health Sciences Research Committee to ask for permission to release the de-identified data

**Reviewers’ Comments**

**Reviewer 1: This is a well-conducted study and addresses an important research question. The manuscript is well-written. I have a few questions for the authors:**

**1. Was propensity matching considered for the analysis of outcomes?**

***Response***

Thank you for the thoughtful question. We considered propensity matching but believe that its merits do not outweigh those of logistic regression and cox regression models to control for the effect of potential confounders. Where outcomes of interest measure conditional treatment effects, propensity matching methods may produce similarly or more biased results compared to regression models (1,2).

**2. Men were more likely to default than women. Could the authors address this finding in the discussion?**

***Response***

Thank you for this suggestion. We have inserted the text below in the discussion to address this.

*Our findings showed that men had lower retention in comparison to women, in line with earlier findings that men have poorer outcomes across the HIV care cascade in sub-Saharan Africa (13,17). Social norms of masculine physical strength, self-reliance and beliefs that clinics are women’s spaces are some reasons for men’s lower retention in HIV services (18,19),* **line 244**

**3. The authors have looked at costs at the individual level, Is it possible to comment on the budgetary / policy implications of these additional costs?**

***Response***

Thank you for your suggestion. We have now inserted a sentence in the discussion to address this.

*“**Considering the difference in costs for the health system between CAD and facility-based care, we estimate that scaling up CAD services by 20% in Malawi would increase the budget of HIV services for 935,000 individuals on ART in Malawi at the end of 2022 by 1,870,000 USD per year (935,000x 20% x 10 USD).”* **line 261**

**4. On what basis was the sample of 700 individuals chosen?**

***Response***

We used an opportunity sample. The sample size in this analysis was based on the total number of people available in the CAD registers, enrolled between January 2019 (when CAD implementation began) and June 2019, to allow for enough for enough follow up at the time of data collection. We then added an equal number of matched individuals on ART at the hub clinics. This has been mentioned in the manuscript, **line 92**.

**Reviewer 2: This is an excellent piece of work. It holds significant importance in the field of HIV/AIDS management and healthcare delivery in resource-limited settings. This study examines the impact of a community-based approach to antiretroviral therapy (ART) distribution in Malawi and evaluates its effectiveness in terms of retention in care, viral load suppression, and cost implications. I would like to congratulate the authors on this effort. The manuscript is organized and well-written. I have only two minor comments

1) Study setting: Add a few lines on disease burden in the two districts of Malawi.**

***Response***

Thank you for this suggestion. We have inserted a sentence in the methods under study setting to address this.

“*Recent population-based estimates indicate that HIV prevalence is 9.0% in Lilongwe city and 13.3% in southwestern Malawi (where Chikwawa is located)*.” line **78**

**2) Table 5: Please define what is meant by ‘resources’ in line 200**

***Response***

Thank you for your question. Table 5 describes ART services usage per client for both CAD and facility-based care. We have changed the wording to make it clearer. **Line 203 and 206**

**Reviewer 3: Thank you for the opportunity to review “Provider-led community Antiretroviral Therapy distribution in Malawi: Retrospective cohort study of retention, viral load suppression, and costs.” This is a well-written manuscript with important implications. The following are a few suggestions for the authors to consider.**

**1) The definition of retention is missing from the methods. Based on the KM curves, it looks like clients were only at risk of becoming lost if they remained in care for at least 2 months. Please confirm this definition. It seems odd that no one died or was transferred within the first 2 months.**

***Response***

We agree that a definition of retention was missing and have inserted this in the Methods, under Key study definitions, line **119:**

**“***Clients who did not experience the adverse outcome are classified as retained after censoring for transfer outs”*

All clients were at risk of the outcomes stated by the reviewer from the day of enrolment. 1 person and 2 people experienced the adverse outcome within the first month and the second month respectively. We have uploaded new figures with improved quality and hope that these details are better reflected.

**2) A table with the univariate and multivariate logistic regression models would be beneficial to include.**

***Response***

This suggestion may indeed be useful for readers. We have included two tables as appendices.

**3) Line 136, change “crude” to unadjusted**

***Response***

We have made the suggested change

**4) Table 5. Resource utilization of CAD-based care and facility-based care in Malawi instead of reporting average and standard it may be more informative to report median (IQR)**

***Response***

In line with reporting of similar articles (3,4), and given that the data are not skewed, we have decided to report mean/SD rather than median/IQR.

**5) Authors should elaborate on the limitations of their study. There is a possibility of a selection bias as well based on the eligibility criteria of the program.**

***Response***

We agree with the potential of bias and had pointed this out, in particular for selection bias, in **line 273:**

“*As is common with observational studies, our results may be prone to bias as clients enrolled in CAD are selected based on specific characteristics, such as being clinically stable, which cannot be completely adjusted for in statistical analyses*”.

We discussed further limitations in **lines 265-272**.

**5) Authors can also try imputing the missing HIV viral values instead of assuming they are missing completely at random (mice command in STATA).**

***Response***

Thank you for this suggestion. We carried out a chi-square to check test if the viral load data were missing at random in reference to treatment arm (CAD/Hub) and the result shows that this is indeed the case (reported in **line** **147**). For this reason, we feel that there is no need to use multiple imputation for missing viral load data.

**6) Figure legends are missing.**

***Response***

The figure captions are imbedded in the manuscript, **lines 183 - 185.**

**References**

1.	Austin PC, Grootendorst P, Normand SLT, Anderson GM. Conditioning on the propensity score can result in biased estimation of common  measures of treatment effect: a Monte Carlo study. Stat Med. 2007 Feb;26(4):754–68.

2.	Shah BR, Laupacis A, Hux JE, Austin PC. Propensity score methods gave similar results to traditional regression modeling  in observational studies: a systematic review. J Clin Epidemiol. 2005 Jun;58(6):550–9.

3.	Nichols BE, Cele R, Jamieson L, Long LC, Siwale Z, Banda P, et al. Community-based delivery of HIV treatment in Zambia: costs and outcomes. AIDS. 2021;35(2):299–306.

4.	Nichols BE, Offorjebe OA, Cele R, Shaba F, Balakasi K, Chivwara M, et al. Economic evaluation of facility‐based HIV self‐testing among adult outpatients in Malawi. J Int AIDS Soc [Internet]. 2020 Sep 9;23(9):e25612. Available from: https://onlinelibrary.wiley.com/doi/10.1002/jia2.25612
